# Supplementary figures and images for: Contribution and functional connectivity between cerebrum and cerebellum on sub-lexical and lexical-semantic processing of verbs
Source: PLoS One. 2023 Sep 14;18(9):e0291558. doi: 10.1371/journal.pone.0291558 (PMC10501569; doi:10.1371/journal.pone.0291558)

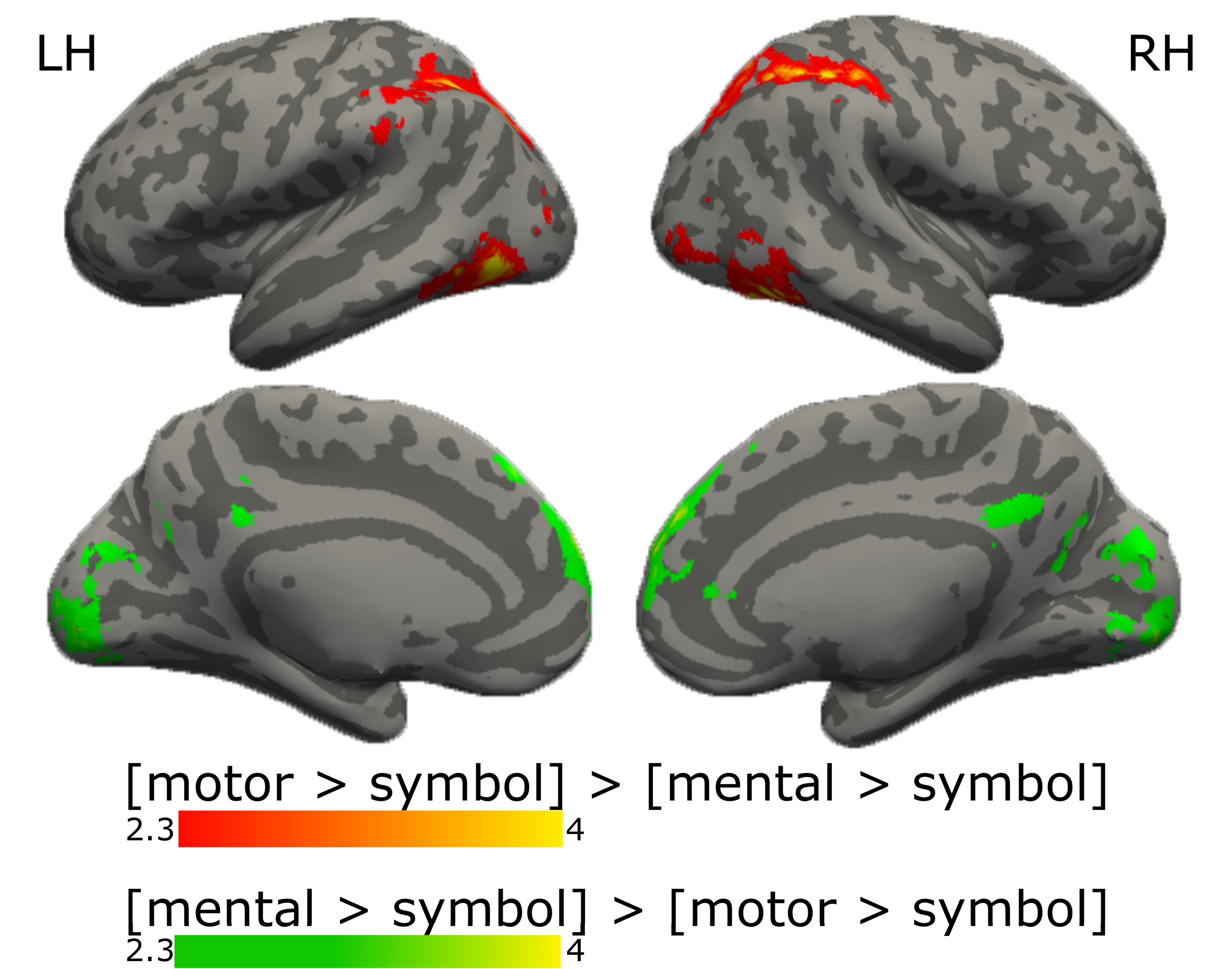

Supplement: S1 Fig — Graphical representation of GLM’s results, brain regions activated in the contrast [motor > symbols] > [mental > symbols] in red, and [mental > symbol] > [motor>symbols] in green. Colorbars show z scores. LH: left hemisphere; RH: right hemisphere. (TIF) [file pone.0291558.s001.tif]
