# Supplementary material for: Contribution and functional connectivity between cerebrum and cerebellum on sub-lexical and lexical-semantic processing of verbs
Source: PLoS One. 2023 Sep 14;18(9):e0291558. doi: 10.1371/journal.pone.0291558 (PMC10501569; doi:10.1371/journal.pone.0291558)
Supplement: S1 Table — The x, y, and z coordinates are in MNI space, regions were labelled according to Harvard-Oxford Cortical and Subcortical Atlases in FSLVIEW. L = Left region or hemisphere. R = Right region or hemisphere. (PDF) [file pone.0291558.s002.pdf]

S1 Table. Brain areas exhibiting significant activation in whole brain analysis during verbs > symbols and pseudo-verbs > symbols contrasts, according to GLM analysis.

Cluster size	Z value	MNI coordinates			Brain region (Harvard Oxford Atlas)
		x	y	z	
**Verbs > Symbols**					
**21080**	**5.47**	**-10**	**50**	**32**	**L Frontal Pole**
	5.14	-58	-30	-4	L Middle Temporal Gyrus, posterior division
	5.08	-20	64	2	L Frontal Pole
	5.01	-52	-4	-18	L Middle Temporal Gyrus, anterior division
	4.95	-12	48	20	L Paracingulate Gyrus
**13226**	**5.78**	**2**	**-76**	**26**	**R Cuneal Cortex**
	5.47	-2	-48	8	L Cingulate Gyrus, posterior division
	5.32	14	-90	20	R Occipital Pole
**3829**	**4.68**	**60**	**-56**	**16**	**R Angular Gyrus**
	4.41	60	-32	-6	R Middle Temporal Gyrus, posterior division
	4.36	66	-50	6	R Middle Temporal Gyrus, temporooccipital part
	4.04	62	-8	10	R Central Opercular Cortex
**Pseudo verbs > Symbols**					
**14935**	**6**	**-54**	**-8**	**38**	**L Precentral Gyrus**
	5.43	-60	-26	-6	L Middle Temporal Gyrus, posterior division
	5.2	-60	-6	-10	L Middle Temporal Gyrus, anterior division
	4.94	-52	-44	12	L Supramarginal Gyrus, posterior division
	4.91	-6	56	38	L Frontal Pole
**1563**	**4.54**	**-4**	**-58**	**4**	**L Lingual Gyrus**
	4.51	-2	-50	6	L Cingulate Gyrus posterior division
	3.26	-6	-70	18	L Supracalcarine Cortex
	2.91	-6	-34	-6	Brain-Stem
	2.9	2	-44	24	R Cingulate Gyrus, posterior division
**865**	**3.78**	**32**	**-86**	**-32**	**R Cerebellum Crus I**
	3.66	14	-90	-30	R Cerebellum Crus II
	3.51	20	-64	-26	R Cerebellum VI
	3.43	24	-68	-54	R Cerebellum VIIIa
	3.07	16	-90	-36	R Cerebellum Crus II

The x, y, and z coordinates are in MNI space, regions were labelled according to Harvard-Oxford Cortical and Subcortical Atlases in FSLVIEW. L = Left region or hemisphere. R = Right region or hemisphere.
