# Supplementary material for: Contribution and functional connectivity between cerebrum and cerebellum on sub-lexical and lexical-semantic processing of verbs
Source: PLoS One. 2023 Sep 14;18(9):e0291558. doi: 10.1371/journal.pone.0291558 (PMC10501569; doi:10.1371/journal.pone.0291558)
Supplement: S2 Table — The x, y, and z coordinates are in MNI space, regions were labelled according to Harvard-Oxford Cortical and Subcortical Atlases in FSLVIEW. L = Left region or hemisphere. R = Right region or hemisphere. (PDF) [file pone.0291558.s003.pdf]

**S2 Table. Brain areas exhibiting significant activation in whole**
**pseudo-verbs > verbs contrasts, according to GLM analysis.**

| Cluster size | Z value | MNI coordinates | | |
|---|---|---|---|---|
| | | x | y | z |
| **Verbs > Pseudo** | | | | |
| **9035** | **4.66** | **-8** | **-26** | **32** |
| | 4.56 | 2 | -16 | 42 |
| | 4.41 | -4 | -40 | 46 |
| **6326** | **4.67** | **20** | **56** | **18** |
| | 4.44 | 4 | 30 | 14 |
| | 4.24 | -18 | 62 | 14 |
| **5768** | **4.25** | **44** | **-58** | **12** |
| | 3.84 | 46 | -76 | 18 |
| | 3.77 | 66 | -18 | 14 |
| | 3.72 | 54 | -62 | 14 |
| **1319** | **3.82** | **-42** | **-62** | **18** |
| | 3.43 | -36 | -52 | 20 |
| | 3.42 | -64 | -56 | 14 |
| | 3.34 | -70 | -52 | 8 |
| **937** | **3.51** | **-18** | **-60** | **-16** |
| | 3.48 | -32 | -36 | -20 |
| | 3.29 | -12 | -68 | -14 |
| | 3.27 | -34 | -16 | -24 |
| **Pseudo verbs >** | | | | |
| **5674** | **5.59** | **-52** | **-4** | **40** |
| | 5.51 | -54 | 4 | 32 |
| | 4.84 | -40 | 28 | 0 |
| **3037** | **4.49** | **-26** | **-68** | **44** |
| | 4.47 | -36 | -44 | 42 |
| | 3.21 | -46 | -34 | 32 |
| | 3.11 | -46 | -50 | 58 |
| **2947** | **4.35** | **-4** | **-78** | **-42** |
| | 4.17 | 24 | -70 | -54 |
| | 4.02 | 26 | -64 | -28 |
| | 3.95 | 40 | -66 | -34 |
| | 3.92 | 6 | -76 | -40 |
| **2591** | **4.82** | **50** | **42** | **18** |
| | 4.5 | 56 | 24 | 24 |
| | 4.25 | 52 | 12 | 24 |
| | 4.14 | 62 | 18 | 24 |
| | 4 | 50 | 32 | 20 |
| **1817** | **4.53** | **28** | **-68** | **50** |
| | 3.47 | 36 | -48 | 42 |
| | 3.05 | 40 | -40 | 40 |
| **1186** | **4.58** | **-42** | **-68** | **-20** |

| | | | | |
|---|---|---|---|---|
| | 4.2 | -44 | -60 | -16 |
| | 2.71 | -44 | -82 | -12 |
| **955** | **4.45** | **0** | **8** | **56** |
| | 3.52 | -14 | 14 | 32 |
| | 2.79 | -14 | 12 | 42 |

The x, y, and z coordinates are in MNI space, regions were label
Subcortical Atlases in FSLVIEW. L = Left region or hemisphere. [illegible]

ᵉ brain analysis during verbs > pseudo verbs and

| ᵒ verbs |
|---|
| **Brain region (Harvard Oxford Atlas)** |
| **L Cingulate Gyrus, posterior division** |
| R Cingulate Gyrus, posterior division, |
| L Precuneous Cortex |
| **R Frontal Pole** |
| R Cingulate Gyrus, anterior division |
| L Frontal Pole |
| **R Middle Temporal Gyrus, temporooccipital part** |
| R Lateral Occipital Cortex, superior division |
| R Planum Temporale |
| R Lateral Occipital Cortex, inferior division |
| **L Lateral Occipital Cortex, superior division** |
| L Supramarginal Gyrus, posterior division, |
| L Angular Gyrus |
| L Middle Temporal Gyrus, temporooccipital part |
| **L Lingual Gyrus** |
| L Temporal Fusiform Cortex, posterior division |
| L Cerebellum VI |
| L Parahippocampal Gyrus, anterior division |
| **Verbs** |
| **L Precentral Gyrus** |
| L Precentral Gyrus |
| L Frontal Orbital Cortex |
| **L Lateral Occipital Cortex, superior division** |
| L Superior Parietal Lobule |
| L Supramarginal Gyrus, anterior division |
| L Supramarginal Gyrus, posterior division |
| **L Cerebellum Crus II** |
| R Cerebellum VIIb |
| R Cerebellum VI |
| R Cerebellum Crus I |
| R Cerebellum Crus II |
| **R Frontal Pole** |
| R Inferior Frontal Gyrus, pars triangularis |
| R Inferior Frontal Gyrus, pars opercularis |
| R Precentral Gyrus, |
| R Middle Frontal Gyrus |
| **R Lateral Occipital Cortex, superior division** |
| R Superior Parietal Lobule |
| R Supramarginal Gyrus, posterior division |
| **L Occipital Fusiform Gyrus** |

| |
|---|
| L Temporal Occipital Fusiform Cortex |
| L Lateral Occipital Cortex, inferior division |
| **Juxtapositional Lobule Cortex (formerly Supplementary Motor Cortex)** |
| L  Cingulate Gyrus, anterior division |
| L Paracingulate Gyrus |

led according to Harvard-Oxford Cortical and
R = Right region or hemisphere.
