# Supplementary material for: Contribution and functional connectivity between cerebrum and cerebellum on sub-lexical and lexical-semantic processing of verbs
Source: PLoS One. 2023 Sep 14;18(9):e0291558. doi: 10.1371/journal.pone.0291558 (PMC10501569; doi:10.1371/journal.pone.0291558)
Supplement: S3 Table — The x, y, and z coordinates are in MNI space, regions were labelled according to Harvard-Oxford Cortical and Subcortical Atlases in FSLVIEW. L = Left region or hemisphere. R = Right region or hemisphere. (PDF) [file pone.0291558.s004.pdf]

S3 Table. Brain areas exhibiting significant activation in whole brain analysis during mental > motor verbs, [mental > symbols] > [motor > symbols] and [mental > pseudo verbs] > [motor > pseudo verbs] contrasts, according to GLM analysis.

Cluster size	Z value	MNI coordinates			Brain region (Harvard Oxford Atlas)
		x	y	z	
**Mental > Motor**					
**3173**	**4.14**	**-14**	**-86**	**-6**	**L Lingual Gyrus**
	4.13	10	-94	-6	R Occipital Pole
	3.88	32	-80	-46	R Cerebellum Crus II
	3.85	14	-74	-22	R Cerebellum VI
	3.43	-12	-98	-4	L Occipital Pole
**1229**	**3.82**	**-60**	**20**	**-2**	**L Inferior Frontal Gyrus, pars opercularis**
	3.78	-50	26	-10	L Frontal Orbital Cortex
	3.71	-60	24	2	L Inferior Frontal Gyrus, pars opercularis
	3.7	-52	26	-6	L Inferior Frontal Gyrus, pars triangularis
**1058**	**3.6**	**-4**	**42**	**48**	**L Superior Frontal Gyrus**
	3.36	0	64	24	Frontal Pole
	3.28	-10	62	26	L Frontal Pole
	3.12	-2	4	64	L Juxtapositional Lobule Cortex (formerly Supplementary Motor Cortex)
	3.06	6	52	38	R Superior Frontal Gyrus
	3.05	0	26	58	Superior Frontal Gyrus
**921**	**3.78**	**-66**	**-30**	**-2**	**L Middle Temporal Gyrus, posterior division**
**[Mental > Symbols] > [Motor > Symbols]**					
**3035**	**3.81**	**8**	**-92**	**-12**	**R Occipital Pole**
	3.54	-6	-94	-6	L Occipital Pole
	3.5	0	-92	-10	Occipital Pole
	3.37	-10	-88	-4	L Lingual Gyrus
**2415**	**3.93**	**2**	**62**	**22**	**R Frontal Pole**
	3.83	-10	62	22	L Frontal Pole
	3.83	6	54	34	R Superior Frontal Gyrus
	3.77	-10	60	26	L Frontal Pole
	3.52	-4	42	46	L Superior Frontal Gyrus
**[Mental > Pseudo verbs] > [Motor > Pseudo verbs]**					
**1344**	**3.53**	**-20**	**-76**	**-10**	**L Occipital Fusiform Gyrus**
	3.37	16	-88	-4	R Lingual Gyrus
	3.22	-8	-90	6	L Occipital Pole
	3.17	14	-90	-8	R Occipital Pole

The x, y, and z coordinates are in MNI space, regions were labelled according to Harvard-Oxford Cortical and Subcortical Atlases in FSLVIEW. L = Left region or hemisphere. R = Right region or hemisphere.
