# Supplementary material for: Contribution and functional connectivity between cerebrum and cerebellum on sub-lexical and lexical-semantic processing of verbs
Source: PLoS One. 2023 Sep 14;18(9):e0291558. doi: 10.1371/journal.pone.0291558 (PMC10501569; doi:10.1371/journal.pone.0291558)
Supplement: S4 Table — The x, y, and z coordinates are in MNI space, regions were labelled according to Harvard-Oxford Cortical and Subcortical Atlases in FSLVIEW. L = Left region or hemisphere. R = Right region or hemisphere. (PDF) [file pone.0291558.s005.pdf]

S4 Table. Brain areas exhibiting significant activation in whole
[mental > symbols] and [mental > symbols] > [motor > symbol

**Cluster size**	**Z value**	**MNI coordinates**		
		**x**	**y**	**z**
**[Motor > Symbols] > [Me**				
**4205**	**4.84**	**-46**	**-66**	**-4**
	4.33	-32	-58	44
	3.93	-28	-70	26
**4184**	**4.6**	**46**	**-56**	**-12**
	4.38	44	-64	-12
	4.19	44	-36	40
	4.06	30	-56	34
	4.05	32	-52	44
**[Mental > Symbols] > [M**				
**3035**	**3.81**	**8**	**-92**	**-12**
	3.54	-6	-94	-6
	3.5	0	-92	-10
	3.37	-10	-88	-4
**2415**	**3.93**	**2**	**62**	**22**
	3.83	-10	62	22
	3.83	6	54	34
	3.52	-4	42	46

The x, y, and z coordinates are in MNI space, regions were label
Subcortical Atlases in FSLVIEW. L = Left region or hemisphere. I

e brain analysis during [motor > symbols] >
s] contrasts, according to GLM analysis.

Brain region (Harvard Oxford Atlas)
**ntal > Symbols]**
**L Lateral Occipital Cortex, inferior division**
L Superior Parietal Lobule
L Lateral Occipital Cortex, superior division
**R Inferior Temporal Gyrus, temporooccipital part**
R Lateral Occipital Cortex, inferior division
R Supramarginal Gyrus, posterior division
R Angular Gyrus
R Superior Parietal Lobule
**otor > Symbols]**
**R Occipital Pole**
L Occipital Pole
Occipital Pole
L Lingual Gyrus
**R Frontal Pole**
L Frontal Pole
R Superior Frontal Gyrus
L Superior Frontal Gyrus

led according to Harvard-Oxford Cortical and
R = Right region or hemisphere.
