# Supplementary material for: Contribution and functional connectivity between cerebrum and cerebellum on sub-lexical and lexical-semantic processing of verbs
Source: PLoS One. 2023 Sep 14;18(9):e0291558. doi: 10.1371/journal.pone.0291558 (PMC10501569; doi:10.1371/journal.pone.0291558)
Supplement: S5 Table — L-SMG = left supramarginal gyrus (-60, -26, 28), R-STG = right superior temporal gyrus (56, -32, 2), L-LOC = left superior lateral occipital cortex (-26, -60, 48), L-MTG = left posterior middle temporal gyrus (-58, -10, -14). EHI = Edinburgh Handedness Inventory index. (PDF) [file pone.0291558.s006.pdf]

S5 Table. Correlation between signal BOLD and behavioral performance.

Contrast	Brain región	Behavioral task	r	p<br>FDR corrected
verbs > symbol	L-SMG	phonological fluency	-0.56	0.019
verbs > pseudoverbs	R-STG	semantic fluency	-0.59	0.011
mental > motor verbs	L-MTG	EHI	-0.54	0.030
	L-LOC	verbs fluency<br>EHI	0.53<br>-0.49	0.034<br>0.034

L-SMG = left supramarginal gyrus (-60, -26, 28), R-STG = right superior temporal gyrus (56, -32, 2), L-LOC = left superior lateral occipital cortex (-26, -60, 48), L-MTG = left posterior middle temporal gyrus (-58, -10, -14). EHI = Edinburgh Handedness Inventory index.
