# Supplementary material for: Contribution and functional connectivity between cerebrum and cerebellum on sub-lexical and lexical-semantic processing of verbs
Source: PLoS One. 2023 Sep 14;18(9):e0291558. doi: 10.1371/journal.pone.0291558 (PMC10501569; doi:10.1371/journal.pone.0291558)
Supplement: S6 Table — The x, y, and z coordinates are in MNI space, regions were labelled according to Harvard-Oxford Cortical and Subcortical Atlases in FSLVIEW. L = Left region or hemisphere. R = Right region or hemisphere. (PDF) [file pone.0291558.s007.pdf]

S6 Table. Brain areas exhibiting significant conectivity during
symbols contrasts, according to PPI analysis with seeds in L-LC

Motor > Sy

PPI seed in le

Cluster size	Z value	MNI coordinates		
		x	y	z
**44129**	**4.96**	**-56**	**-16**	**-12**
	4.79	-58	-14	18
	4.73	-6	-72	18
	4.64	-54	-60	4

Mental > Sy

PPI seed in le

Cluster size	Z value	MNI coordinates		
		x	y	z
**1660**	**3.68**	**-40**	**60**	**0**
	3.59	0	60	4
	3.4	2	54	-2
	3.35	-34	56	2
	3.1	16	66	4
	2.8	8	38	-2
**1339**	**3.35**	**18**	**22**	**60**
	3.16	14	38	30
	3.14	0	26	60
**1037**	**3.6**	**-6**	**-18**	**-4**
	3.47	10	-8	0
	3	16	-28	-10

PPI seed in right

**5032**	**3.64**	**0**	**-72**	**14**
	3.61	-4	-90	10
	3.53	4	-74	0
**2796**	**3.77**	**52**	**-54**	**12**
	3.63	56	-8	-8
	3.62	60	-42	8
	3.59	66	-18	-14
	3.17	54	6	-16
**2161**	**3.62**	**-56**	**-2**	**-16**
	3.6	-52	-16	14
	3.58	-62	-4	18
	3.19	-58	-18	0
	3.15	-48	-50	6
	3.14	-62	-32	-8
**985**	**3.58**	**16**	**-30**	**72**
	2.99	26	-26	54
**764**	**3.31**	**-8**	**58**	**-2**

	3.07	-10	54	2
	3.01	0	60	6
	2.98	10	54	-4
				**PPI seed in le**
**52888**	**5**	**-52**	**-16**	**12**
	5	-42	-20	58
	4.88	-44	-24	40
				**Pseudo > Sy**
				**PPI seed in le**
**Cluster size**	**Z value**	**MNI coordinates**		
		**x**	**y**	**z**
**3667**	**4.16**	**-52**	**18**	**20**
	4.08	-56	-8	44
	4.02	-52	30	2
				**PPI seed in le**
**1005**	**3.51**	**40**	**38**	**32**
	3.38	42	32	32
	3.16	42	16	6

The x, y, and z coordinates are in MNI space, regions were label
Subcortical Atlases in FSLVIEW. L = Left region or hemisphere. R

motor > symbols, mental > symbols and peudo verbs >
)C, L-MTG, L-SMA and R cerebellum.

ymbol

eft LOC

Brain region (Harvard Oxford Atlas)
**L Middle Temporal Gyrus, posterior division**
L Central Opercular Cortex
L Intracalcarine Cortex
L Middle Temporal Gyrus, temporooccipital part

ymbol

eft SMA

Brain region (Harvard Oxford Atlas)
**L Frontal Pole**
Frontal Pole
R Paracingulate Gyrus
L Frontal Pole
R Frontal Pole
R Cingulate Gyrus, anterior division
**R Superior Frontal Gyrus**
R Paracingulate Gyrus
Superior Frontal Gyrus
**L Thalamus**
R Thalamus
R Parahippocampal Gyrus

cerebellum

**Supracalcarine Cortex**
L Occipital Pole
R Lingual Gyrus
**R Angular Gyrus**
R Superior Temporal Gyrus, posterior division
R Middle Temporal Gyrus, temporooccipital part
R Middle Temporal Gyrus, posterior division
R Temporal Pole
**L Middle Temporal Gyrus, anterior division**
L Central Opercular Cortex
L Precentral Gyrus
L Superior Temporal Gyrus, posterior division
L Middle Temporal Gyrus, temporooccipital part
L Middle Temporal Gyrus, posterior division
**R Postcentral Gyrus**
R Precentral Gyrus
**L Frontal Pole**

L Paracingulate Gyrus
Frontal Pole
R Paracingulate Gyrus
**eft LOC**
**L Central Opercular Cortex**
L Precentral Gyrus
L Postcentral Gyrus
**ymbol**
**eft LOC**
**Brain region (Harvard Oxford Atlas)**
**L Inferior Frontal Gyrus, pars opercularis**
L Precentral Gyrus
L Inferior Frontal Gyrus, pars triangularis
**eft MTG**
**R Frontal Pole**
R Middle Frontal Gyrus
R Frontal Operculum Cortex

led according to Harvard-Oxford Cortical and
R = Right region or hemisphere.
