# Supplementary material for: Contribution and functional connectivity between cerebrum and cerebellum on sub-lexical and lexical-semantic processing of verbs
Source: PLoS One. 2023 Sep 14;18(9):e0291558. doi: 10.1371/journal.pone.0291558 (PMC10501569; doi:10.1371/journal.pone.0291558)
Supplement: S7 Table — The x, y, and z coordinates are in MNI space, regions were labelled according to Harvard-Oxford Cortical and Subcortical Atlases in FSLVIEW. L = Left region or hemisphere. R = Right region or hemisphere. (PDF) [file pone.0291558.s008.pdf]

S7 Table. Brain areas exhibiting significant connectivity durin
according to PPI analysis with seeds in L-LOC, L-SMA and R cer

Motor > Ps

PPI seed in le

Cluster size	Z value	MNI coordinates		
		x	y	z
**23866**	**4.47**	**66**	**-22**	**18**
	4.27	62	-28	16
	4.2	-52	-26	6
	4.18	66	-18	16
	4.14	10	-60	12

PPI seed in le

Cluster size	Z value	MNI coordinates		
		x	y	z
**6050**	**4.06**	**62**	**-34**	**22**
	3.9	58	-38	14
	3.86	54	-36	18
	3.84	52	-2	-30
	3.71	44	-18	22
**3636**	**3.75**	**-42**	**-4**	**-4**
	3.56	-56	-28	12
	3.52	-50	0	-32
	3.46	-46	-26	8
**2753**	**3.38**	**-14**	**-46**	**30**
	3.32	-34	-16	56
	3.26	-6	-48	40
**1393**	**3.72**	**-16**	**58**	**12**
	3.21	12	58	24

Mental > Ps

PPI seed in le

Cluster size	Z value	MNI coordinates		
		x	y	z
**21949**	**4.7**	**64**	**-16**	**18**
	4.36	-26	-40	62
	4.34	60	-10	14
	4.24	-54	-24	10
	4.04	64	-32	26
	3.96	64	-26	22

PPI seed in le

Cluster size	Z value	MNI coordinates		
		x	y	z
**16877**	**4.48**	**2**	**-46**	**14**
	4.2	-6	-28	38
	4.14	8	-58	28

	4.12	66	-34	24
**8164**	**4.54**	**0**	**54**	**-2**
	4.42	-20	48	26
	4.29	-8	48	8
**1218**	**3.72**	**-44**	**-58**	**14**
	3.33	-46	-56	4
	3.29	-46	-78	22
	3.14	-56	-50	12
**PPI seed in right c**				
**Cluster size**	**Z value**	**MNI coordinates**		
		**x**	**y**	**z**
**4925**	**3.94**	**56**	**-10**	**-10**
	3.8	56	-56	10
	3.71	50	-8	24
	3.67	56	-40	8
**2932**	**3.66**	**32**	**-30**	**70**
**2360**	**3.57**	**-14**	**38**	**48**
	3.42	-2	48	24
	3.28	-18	48	38
	3.15	4	20	58
**2325**	**3.89**	**-58**	**-44**	**-10**
	3.17	-42	-78	20
	3.17	-52	-38	-12
	3.13	-62	-10	-12
	3.11	-50	-22	10
**1194**	**3.33**	**14**	**-58**	**32**
	3.27	-6	-80	32
	3.11	-16	-48	30
	3.01	-6	-60	46
	2.94	-8	-86	12

The x, y, and z coordinates are in MNI space, regions were label
Subcortical Atlases in FSLVIEW. L = Left region or hemisphere. I

g motor > pseudo verbs and mental > pseudo verbs ,
·ebellum.

eudo

·ft LOC

Brain region (Harvard Oxford Atlas)
**R Supramarginal Gyrus, anterior division**
R Planum Temporale
L Planum Temporale
R Postcentral Gyrus
R Precuneous Cortex

ft SMA

Brain region (Harvard Oxford Atlas)
**R Parietal Operculum Cortex**
R Supramarginal Gyrus, posterior division
R Planum Temporale
R Middle Temporal Gyrus, anterior division
R Central Opercular Cortex
**L Insular Cortex**
L Planum Temporale
L Middle Temporal Gyrus, anterior division
L Heschl's Gyrus (includes H1 and H2)
**L Cingulate Gyrus, posterior division**
L Precentral Gyrus
L Precuneous Cortex
**L Frontal Pole**
R Frontal Pole

;eudo

·ft LOC

Brain region (Harvard Oxford Atlas)
**R Postcentral Gyrus**
L Postcentral Gyrus
R Central Opercular Cortex
L Planum Temporale
R Parietal Operculum Cortex
R Supramarginal Gyrus, anterior division

ft SMA

Brain region (Harvard Oxford Atlas)
**R Cingulate Gyrus, posterior division**
L Cingulate Gyrus, posterior division
R Precuneous Cortex

R Superior Temporal Gyrus, posterior division
**Paracingulate Gyrus**
L Frontal Pole
L Paracingulate Gyrus
**L Angular Gyrus**
L Middle Temporal Gyrus, temporooccipital part
L Lateral Occipital Cortex, superior division
L Supramarginal Gyrus, posterior division

erebellum

Brain region (Harvard Oxford Atlas)
**R Superior Temporal Gyrus, posterior division**
R Middle Temporal Gyrus, temporooccipital part
R Postcentral Gyrus
R Supramarginal Gyrus, posterior division
**R Postcentral Gyrus**
**L Superior Frontal Gyrus**
L Paracingulate Gyrus
L Frontal Pole
R Superior Frontal Gyrus
**L Middle Temporal Gyrus, temporooccipital part**
L Lateral Occipital Cortex, superior division
L Middle Temporal Gyrus, posterior division
L Middle Temporal Gyrus, anterior division
L Heschl's Gyrus (includes H1 and H2)
**R Precuneous Cortex**
L Cuneal Cortex
L Cingulate Gyrus, posterior division
L Precuneous Cortex
L Intracalcarine Cortex

led according to Harvard-Oxford Cortical and
R = Right region or hemisphere.
