# Supplementary material for: Contribution and functional connectivity between cerebrum and cerebellum on sub-lexical and lexical-semantic processing of verbs
Source: PLoS One. 2023 Sep 14;18(9):e0291558. doi: 10.1371/journal.pone.0291558 (PMC10501569; doi:10.1371/journal.pone.0291558)
Supplement: S8 Table — The x, y, and z coordinates are in MNI space, regions were labelled according to Harvard-Oxford Cortical and Subcortical Atlases in FSLVIEW. L = Left region or hemisphere. R = Right region or hemisphere. (PDF) [file pone.0291558.s009.pdf]

S8 Table. Brain areas exhibiting significant connectivity during mental > motor verbs according to PPI analysis with seeds in right cerebellum.

Mental > Motor					
PPI seed in right cerebellum					
Cluster size	Z value	MNI coordinates			Brain region (Harvard Oxford Atlas)
		x	y	z	
**1181**	**3.7**	**-28**	**-84**	**-2**	**L Lateral Occipital Cortex, inferior division**
	3.05	4	-96	14	R Occipital Pole
	3.03	-20	-84	14	L Lateral Occipital Cortex, superior division
	3	-16	-84	10	L Intracalcarine Cortex
	2.98	10	-80	34	R Cuneal Cortex

The x, y, and z coordinates are in MNI space, regions were labelled according to Harvard-Oxford Cortical and Subcortical Atlases in FSLVIEW. L = Left region or hemisphere. R = Right region or hemisphere.
